# EP02 A rare case of Reactive arthritis secondary to COVID-19

**DOI:** 10.1093/rap/rkaa052.001

**Published:** 2020-11-03

**Authors:** Arslan Sidhu, Shilpa Selvan, Ziad Alkutobi

**Affiliations:** Basildon and Thurrock University Hospitals, Basildon, United Kingdom

## Abstract

**Case report - Introduction:**

In December 2019, the first cluster of Coronavirus disease 2019 (COVID-19) cases caused by the novel coronavirus SARS-CoV-2 was identified in Wuhan, China. The disease was declared a global pandemic on 11^th^ March 2020. COVID-19 was initially thought to cause respiratory complications only, however several extra pulmonary manifestations of the infection have since emerged.

We report a rare case of reactive arthritis (ReA), urticarial rash and angioedema in a young female secondary to COVID-19 infection. Rashes were recently added to the World Health Organisation (WHO) criteria for diagnosis of COVID-19 demonstrating their significance.

**Case report - Case description:**

A 31-year-old female doctor was admitted with acute swelling of her lips, dysphagia, and a widespread urticarial rash. Preceding this she had a one-week history of fever, cough, and constitutional symptoms of malaise and weight loss. Her symptoms had started at the end of April 2020 during the peak of the COVID-19 pandemic in the United Kingdom. Three days later she developed painful swelling of her wrists, elbows, knees, and hands. She reported no back or sacroiliac joint pain, enthesitis or any previous history of inflammatory joint pains. She had a history of platelet dysfunction and was treated with Desmopressin.

Clinical examination revealed a widespread urticarial rash over her face, limbs, and trunk, with no nail abnormalities. She had active synovitis in her right wrist, elbow, and mild bilateral knee effusions. All other joints including spine and sacroiliac joints were normal. She had no dactylitis or enthesitis. Systemic examination was normal. Investigations revealed Hb 113 g/L, MCV 88.2 fL, Platelets 282 x 10^9^/L, WCC 6.6 x 10^9^/L and Lymphocytes of 0.63 x 10^9^/L with normal neutrophil and eosinophil count. CRP was raised at 107mg/L. She had a negative autoimmune screen including ANA, ANCA, IgM-RF, anti-CCP antibodies and HLA B27. Plain radiographs of knees were normal. SARS CoV-2 PCR was positive following a nasal swab. Urine and blood cultures were negative. Treatment was commenced with intravenous hydrocortisone and antihistamines with resolution of her angioedema symptoms; however, her rash and arthritis persisted.

The patient was diagnosed with Reactive Arthritis (ReA), urticarial rash and angioedema secondary to COVID-19 infection. Prednisolone 30mg daily was started, and within a week her arthritis and rash markedly improved. Prednisolone was tapered over six weeks. By her two-month clinic follow up, she reported no further joint swelling and was functioning normally.

**Case report - Discussion:**

The most common complication of COVID-19 is Acute Respiratory Distress Syndrome (ARDS) however several other serious complications have been identified including cardiac injury, thromboembolic events, neurological abnormalities, and an aggravated inflammatory response causing a cytokine storm.

ReA is a post infectious arthritis commonly seen following gastrointestinal or genitourinary infections and is yet to be recognised as a complication of this disease. ReA most commonly presents as an asymmetrical peripheral or axial spondyloarthropathy. The affected joints do not contain pathogen. More than half of ReA cases resolve spontaneously within six months without requiring long-term treatments.

Up to 20% of patients with COVID-19 infection have been shown to develop cutaneous manifestations including erythematous rash, vesicular rash, acral ischaemia, rash with petechiae, and widespread urticaria. This has led to the recent addition of rashes to the World Health Organisation (WHO) Criteria for diagnosis of COVID-19 infection. Additionally, as COVID-19 has an incubation period of 14 days where patients can be asymptomatic, cutaneous manifestations may serve as an early indicator of infection, aiding in a more rapid diagnosis.

**Case report - Key learning points:**

We present a rare case of ReA secondary to COVID-19 infection, with complete resolution of symptoms following administration of oral glucocorticoids. A detailed history and examination of the musculoskeletal system should be undertaken in all patients presenting with COVID-19. Urticarial rashes should be considered as an early symptom of COVID-19 infection as per the WHO criteria for diagnosis. Glucocorticoids can be considered in treating patients with this presentation, where traditional anti-inflammatory agents have been refractory or contraindicated.

